# Fatty Acid-Derived *N*-acylethanolamines Dietary Supplementation Attenuates Neuroinflammation and Cognitive Impairment in LPS Murine Model

**DOI:** 10.3390/nu14183879

**Published:** 2022-09-19

**Authors:** Anna Tyrtyshnaia, Sophia Konovalova, Arina Ponomarenko, Anastasia Egoraeva, Igor Manzhulo

**Affiliations:** A.V. Zhirmunsky National Scientific Center of Marine Biology, Far Eastern Branch, Russian Academy of Sciences, Palchevskogo Str. 17, 690041 Vladivostok, Russia

**Keywords:** *N*-acylethanolamine, NAE, fatty acid, DHEA, EPEA, SEA, OEA, PEA, dietary supplement, neuroinflammation, hippocampus, neurogenesis, memory

## Abstract

Neuroinflammation plays a critical role in the pathogenesis of most neurological and neurodegenerative diseases and therefore represents a potential therapeutic target. In this regard, accelerating the resolution process in chronic neuroinflammation may be an effective strategy to deal with the cognitive consequences of neuropathology and generalized inflammatory processes. *N*-acylethanolamine (NAE) derivatives of fatty acids, being highly active lipid mediators, possess pro-resolving activity in inflammatory processes and are promising agents for the suppression of neuroinflammation and its consequences. This paper is devoted to a study of the effects played by dietary supplement (DS), containing a composition of fatty acid-derived NAEs, obtained from squid *Berryteuthis magister*, on the hippocampal neuroinflammatory and memory processes. By detecting the production of pro-inflammatory cytokines and glial markers, a pronounced anti-inflammatory activity of DS was demonstrated both in vitro and in vivo. DS administration reversed the LPS-induced reduction in hippocampal neurogenesis and memory deterioration. LC-MS analysis revealed an increase in the production of a range of NAEs with well-documented anti-inflammatory activity in response to the administered lipid composition. To conclude, we found that tested DS suppresses the neuroinflammatory response by reducing glial activation, positively regulates neural progenitor proliferation, and attenuates hippocampal-dependent memory impairment.

## 1. Introduction

**Figure 1:**
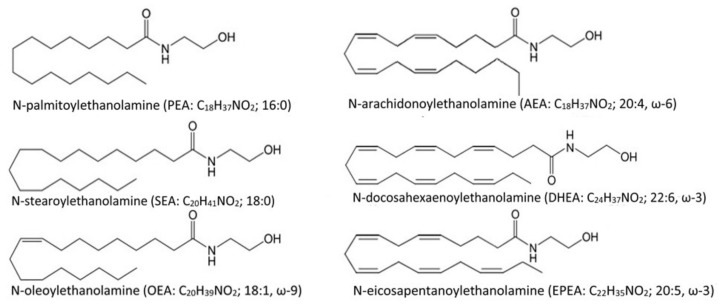
Fatty acid-derived *N*-acylethanolamines.

Neuroinflammation is a condition characterized by glial activation and the production of a wide range of pro-inflammatory mediators in the central nervous system. A large proportion of neurobiological research is devoted to the study of neuroinflammation since the neuroinflammatory response is characteristic of most neurological and neurodegenerative diseases [1,2]. In addition, neuroinflammation can also develop in the case of systemic infections [3], causing long-term disability and creating a need for rehabilitation measures. Like peripheral inflammation, neuroinflammation plays a dual role: on the one hand, it performs a protective function, limiting the focus of damage or contributing to the destruction of the pathogen, on the other hand, it leads to neuronal death and neurodegeneration. In particular, activated microglia can secrete both pro-inflammatory cytokines, leading to further damage and anti-inflammatory factors that promote regeneration [4]. For example, pro-inflammatory cytokines such as IL-1β, IL-6, and tumor necrosis factor-a (TNF-α) induce neuronal death [5]. In addition, these factors increase the production of glutamate in neurons, enhancing neuronal excitotoxicity through the activation of NMDA receptors [6]. At the same time, mice deficient in the anti-inflammatory cytokine IL-10 show a more pronounced cognitive deficit in LPS-induced neuroinflammation [7]. The activity of anti-inflammatory factors aims at limiting the severity and duration of the inflammatory response and provides resolution of neuroinflammation in an acute process. A pathological condition develops when the resolution of the inflammation is impossible [8]. In this case, neuroinflammation becomes chronic and is maintained by exposure to positive feedback. In this regard, accelerating the resolution process in chronic neuroinflammation may be an effective strategy to deal with the cognitive consequences of generalized inflammatory processes. Promising in this case are strategies aimed at stimulating the production of pro-resolving lipid mediators or exogenous administration of these compounds. There are several groups of lipid mediators with anti-inflammatory activity, which include n-6 fatty acids derivatives: AA-derived lipoxins, as well as omega-3 fatty acid derivatives: EPA-derived and DHA-derived resolvins, protectins, and maresins [9]. A special highly active group of anti-inflammatory lipid mediators is non-oxidative *N*-acylethanolamine (NAE) derivatives of fatty acids [10], which include *N*-docosahexaenoylethanolamine (DHEA), *N*-eicosapentanoylethanolamine (EPEA), *N*-stearoylethanolamine (SEA), *N*-palmitoylethanolamine (PEA), *N*-oleoylethanolamine (OEA), *N*-arachidonoylethanolamine (AEA), etc. (Figure 1).

The mechanisms of n-6 arachidonic acid metabolite AEA (anandamide) action on the central nervous system are mediated mainly by an affinity for CB receptors, and therefore the substance belongs to the class of endocannabinoids along with 2-arachidonoyl glycerol [11]. The activity of other fatty acids ethanolamides is mainly realized through cannabinoid-independent pathways. These pathways include activation of nuclear peroxisome proliferator-activated receptor-α (PPAR-α), suppression of NF-kB and ERK1/2-dependent signaling pathway, and accumulation of cAMP followed by phosphorylation of protein kinase A (PKA) and the cAMP response element binding protein (CREB) [12,13,14,15,16]. The biological activity of NAE is not limited to anti-inflammatory properties. For example, in addition to anti-inflammatory [17], PEA possesses analgesic activity, which is partially realized through CB2 receptors [18] and antiepileptic potential [19]. OEA regulates energy metabolism and has an anorexigenic effect [20,21]. DHEA (synaptamide) is involved in brain development and function by promoting synaptogenesis, neurogenesis, and neuritogenesis [21,22,23]. Not much is known about SEA, for example, it is an inhibitor of the sphingolipid signaling pathway and blocks the effect of TNF- and arachidonic acid on intracellular Ca^2+^ concentration, having neuroprotective activity [24]. There are also data on the anti-inflammatory activity of EPEA, which is slightly inferior to that of DHEA [25,26]. The sources of NAE are very diverse: some of them are found in plants, and some can only be obtained from animal sources. For example, corn, tomatoes, peanuts, soybeans, and cotton seeds can serve as a food source for PEA [27]. Plant foods with the highest OEA content are wheat flour, cocoa, and coffee [28]. However, compared to PEA, the OEA content of animal products is higher than that of plant products [28]. AEA is not found in plant foods at all, except for cocoa, and its source is predominantly eggs, chicken, and beef [28]. Animal products can also serve as potential sources of DHEA and EPEA, although their content in various sources has not been studied in detail. In mammals, these important signaling lipids are predominantly synthesized from exogenous DHA and EPA [25,29]. Food is the main source of DHA and EPA for the body, although these essential PUFAs can also be formed by lengthening the acyl chain and forming additional double bonds of α-linolenic acid (18:3n-3, ALA), then converting to EPA (20:5n-3) through the intermediate stearidonic acid (18:4n-3). Further, EPA can be metabolized to docosapentaenoic acid (22:5n-3; DPA) and finally to DHA (22:6n-3). However, the endogenous conversion of ALA to EPA and DHA is very limited in humans, especially adults, and occurs only under conditions of deficiency [30]. A number of studies demonstrate that dietary enrichment with DHA and EPA results in increased tissue concentrations of corresponding ethanolamines [10,31,32,33]. Oily fish are the richest sources of DHA, DPA, and EPA, but they are also found in significant amounts in other marine organisms, including algae [34].

In our work, by-products of squid *Berryteuthis magister* processing served as a source of PUFA for NAE synthesis. As a rule, during the industrial processing of squid, internal organs are discarded or used for feed production; however, they contain a high concentration of fat and are a valuable source of nutrients. For example, squid liver contains about 35% of the lipid fraction, of which 13% is fatty acids [35]. We decided to use this source to obtain a composition of PUFA ethanolamides, including DHEA, EPEA, and SEA.

This work is devoted to the study of the effect of the dietary supplement (DS), containing a composition of fatty acid ethanolamides, obtained from *Berryteuthis magister*, on the hippocampal neuroinflammatory and memory processes.

## 2. Materials and Methods

### 2.1. Cell Culture

For in vitro studies, we used a microglial cell line SIM-A9 (T0247-GVO-ABM, BioCat, Heidelberg, Germany). Mouse microglial cells were cultured in 24-well microplates at a density of 1 × 105 cells per cm^2^ in complete DMEM/F12 medium and incubated for 1 h at 37 °C with 5% CO_2_. After cell adhesion, the culture medium was replaced with a medium containing the test DS (composition of *N*-acylethanolamines) in 0.1, 1, and 10 μg/mL concentrations and incubated at 37 °C with 5% CO_2_ for one hour. Next, the solution of bacterial lipopolysaccharides (LPS, E. coli O111:B4, Sigma-Aldrich, Bellefonte, PA, USA) was added to the samples to obtain a concentration of 1 μg/mL and incubated at 37 °C with 5% CO_2_ for 24 h. The experiment used a negative control (cells incubated in a normal culture medium poor DS) and control of LPS activity (cells incubated with LPS).

### 2.2. Animals and Treatment

The study used male mice of the C57BL/6 line (3 months old), which were grown in the vivarium of the A.V. Zhirmunsky National Scientific Center for Marine Biology, Far Eastern Branch of the Russian Academy of Sciences, Vladivostok, Russia. There were 3–4 mice in a cage. A 12 h light/dark cycle was maintained with a temperature of 23 ± 2 °C and humidity of 55 ± 15%. Animals had ad libitum access to food and water. All experimental methods were approved by the Animal Ethics Committee of the National Scientific Center for Marine Biology, Far Eastern Branch of the Russian Academy of Sciences (No. 1.3/2022) following the Guidelines for the Welfare of Laboratory Animals and the Directive of the Council of the European Community 2010/63/EC.

To induce neuroinflammation, systemic administration (intraperitoneally, i.p.) of bacterial lipopolysaccharides (LPS, E. coli O111:B4, Sigma-Aldrich) at a dose of 700 µg/kg for two consecutive days was used. The studied DS was administered orally at a dose of 10 mg/kg. The study involved four groups of mice: “Veh” (*n* = 26)—the i.p. injection of saline and oral water administration; “LPS” (*n* = 26)—i.p. injection of LPS and oral water administration; “LPS + DS” (*n* = 26)—i.p. injection of LPS and oral DS administration; “Veh + DS” (*n* = 26)—the i.p. injection of saline and oral DS administration. Injections of saline or LPS (700 mg/kg) were administered for two consecutive days. The volume of injected substances was 100 µL. Oral administration of DS emulsion using a gavage procedure began simultaneously with the LPS treatment but continued for 7 days. The dietary supplement was emulsified by continuous shaking on a Multi-Vortex shaker (V-32, Biosan, Riga, Latvia).

### 2.3. Dietary Supplement Preparation

**Figure 2:**
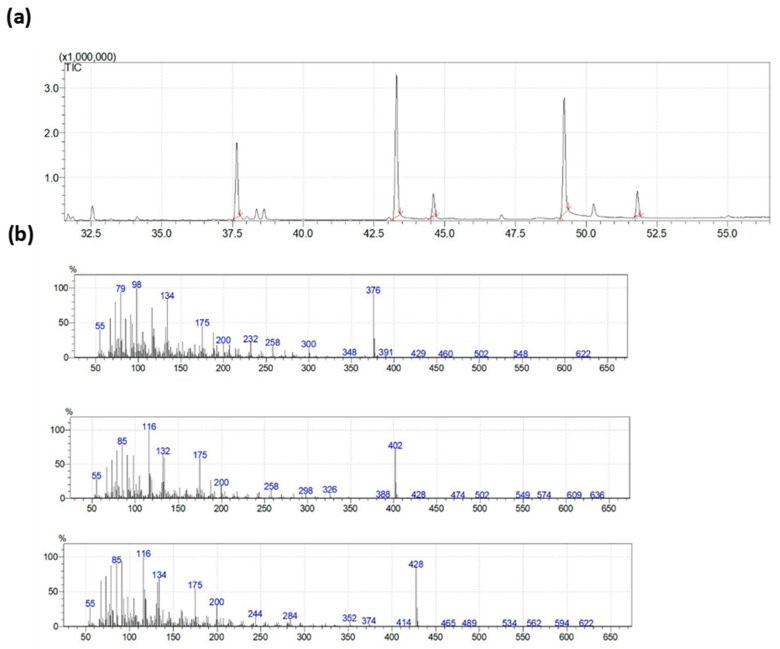
Total ion current chromatogram and electron ionization mass spectra of PUFA ethanolamides trimethyl silyl derivatives included in the composition under study. (**a**) Total ion current chromatogram of PUFA ethanolamides trimethyl silyl derivatives. (**b**) electron ionization mass spectra of *N*-stearoylethanolamine trimethylsilyl derivatives (**top**), *N*-eicosapentanoylethanolamine (**middle**) and *N*-docosahexanoylethanolamine (**bottom**).

The concentrate of polyunsaturated fatty acids (PUFA) was isolated from the processing waste of squid *Berryteuthis magister*, caught at a depth of 400–600 m in the western part of the Bering Sea, according to the Latyshev method [36]. The composition of PUFA ethanolamides was determined by converting them to trimethylsilyl derivatives (TMS-NAE). For conversion, we added 50 µL of N,O-bis(trimethylsilyl)trifluoroacetamide (BSTFA) to 1 mg of fatty acid ethanolamides, and the mixture was heated to 60 °C for 1 h in an argon atmosphere. To quantitatively determine the PUFA ethanolamides’ composition, we added 1 mL of hexane and then 1 µL of each silylated fraction was analyzed using the gas chromatography (GC) system. The mixture separation was performed using the following conditions: (1) initial temperature of 180 °C; (2) heating rate from 2 °C/min to 260 °C; and (3) the time of temperature maintenance was 35 min. The temperatures of the injector and detector were set to 260 °C. A Shimadzu GC-2010 plus chromatograph was used with a Supelco SLB™—5 ms, 30 m × 0.25 mm capillary column (Sigma-Aldrich) and a flame ionization detector (Shimadzu, Kyoto, Japan). A gas chromato-mass spectrometer (GC-MS) was used to identify TMS-NAE. Mass spectra were obtained using a Shimadzu TQ-8040 equipment (Shimadzu) with a Supelco SLB™—5 ms column (Sigma-Aldrich). Mass spectra of trimethylsilyl derivatives of PUFA ethanolamides recorded by GC-MS are given in Figure 2.

### 2.4. Western Blotting

Western blotting was used to determine INFγ, TNFα, IL-1β, IL6, and MHCII levels in mouse microglial cells SIM-A9 treated with LPS and DS. Cells were detached from the plate with Accutase™ Cell Detachment Solution (561527, BD Biosciences, New Jersey, USA) and 1 mL of saline was added to the samples. Detached cells were ultrasonically homogenized in dilution buffer (PBS) supplemented with 0.150 mM serine protease inhibitor (PMSF). The final protein concentration was adjusted to 2 mg/mL. Samples were then diluted 1:1 with standard loading buffer (1x sample buffer, Biorad, Hercules, CA, USA) containing 5% 2-mercaptoethanol, placed in a water bath at 94.5 °C, and incubated for 5 min. Electrophoresis was performed using a Biorad system using Protean mini gel Any kDa gel cartridges (Biorad) and Spectra Multicolor Broad Range Protein Ladder (Thermo Fisher Scientific, Waltham, MA, USA). The well loading was 60 μg of protein, and the current per gel was 15 mA with a constant voltage of 120 V. After electrophoresis, proteins were transferred to a PVDF membrane using a Turbo transblot transfer system (Biorad). All transfer materials were used from the Transblot Turbo RTA Transfer Kit (Biorad). The membranes were then placed in blocking buffer (1x phosphate buffer containing 2% BSA, 0.1% Tween20, 0.05% Triton X-100) overnight. The next day, the blocking buffer was washed with PBS + 0.1% Tween20 and then incubated for 1 h with primary antibodies: anti-Interferon gamma antibody (1:1000, ab52746), anti-TNFα (1:1000, ab208348), anti-IL-1β (1:1000, ab205924), anti-IL6 (1:1000, ab46100) and anti-MHCII (1:500, ab23990), all from Abcam (Cambridge, UK). β-tubulin (1:500, ab6046) was used as a loading control. After incubation with primary antibodies, the membranes were washed again with PBS-T, then incubated for an hour with secondary antibodies Anti-Rabbit (Abcam) and Anti-Mouse (Abcam). Western Blot ECL Substrate (Biorad) was used to carry out the chemiluminescence reaction (1 mL of substrate per membrane, 5 min). Visualization was performed using the ChemiDoc gel documentation system (Biorad). The resulting images were analyzed using the ImageLab software package (Biorad).

### 2.5. Nitrite Quantification

Nitrite accumulated in the culture medium after incubation with LPS and DS was measured using the Griess method. To do this, 100 µL of Griess reagents were added to 100 µL of cell culture medium and incubated at room temperature for 10 min. Absorbance was determined at 540 nm using an iMark plate spectrophotometer (Biorad). The results were presented as a percentage of the negative control.

### 2.6. N-acylethanolamines Quantification in Brain Lipids

For the experiment, a separate cohort of 32 mice was used, which received DS for 7 consecutive days in doses 10, 20, and 40 mg/kg. For *N*-acylethanolamines analysis, animals were deeply anesthetized with isoflurane, and brains were quickly removed, divided into 2 hemispheres, frozen in liquid nitrogen, and stored at −80 °C until use. After the addition of 0.1 nmol internal standard 22:0-NAE to the frozen mouse brain, lipid extraction was performed according to the method of Bligh and Dyer (1959) [37] Ice-cold CHCl3 (1 mL) was used to restore the final lipid residue. The lipid solution was purged with argon and stored at −80 °C until liquid chromatography-mass spectrometry (LC-MS) was performed.

An Ascentis C18 analytical column (2.1 mm × 100 mm × 3-micron, Supelco, Bellefonte, PA, USA) and LC-MS 8060 (Shimadzu) equipped with an atmospheric-pressure-heated electrospray ionization source in positive polarity mode were used for NAE LC-MS analysis. Multiple reaction monitoring (MRM) was performed for the mixture analysis. The following ion source parameters were used: 380 °C—interface temperature; 250 °C—desolvation line temperature; 3 L/min—nebulizing gas (N2) flow; 3 L/min—drying gas (N2) flow; and 17 L/min—heating gas (dry air) flow. The collision energy was optimized individually for each compound. For each measured compound, the following molecular ion and fragment were set: 348 → 62 for AEA, 372 → 62 for DHEA, 326 → 62 for OEA, 300 → 62 for PEA, and 384 → 62 for the internal standard (22:0-NAE). To quantify each NAE in the tissue sample, LabSolution (Shimadzu) was used. The comparison of the peak area with the internal standard peak area was performed and the values were expressed as nmoL/g.

### 2.7. Behavioral Studies

#### 2.7.1. Y-Maze Testing

The working memory of animals was evaluated in a Y-maze using a spontaneous alternation test. The Y-maze consisted of three equal arms with the following parameters: 30 cm in length, 40 cm in height, and 10 cm in width. Each animal was placed in the center of the maze and left for 5 min to explore the test space freely. The criterion for arm entering was the placement of all 4 paws inside the arm. Spontaneous alternation rate (SAR) was calculated using the following formula: R (%) = Mx100/(N-2), where N—total number of entries, and M—number of “correct” triples (consecutive selections of each of the 3 arms without repeated entries). The total number of arm entrances for each mouse was used to determine locomotor activity.

#### 2.7.2. Novel Object Recognition Test

The novel object recognition (NOR) test was performed according to the method of Bevins and Beshear [38]. The day before testing, the animals were placed in a test apparatus for familiarization and habituation (pre-training). In the first stage (training session), each mouse was placed in the center of the arena with two identical objects located on both sides, allowing them to explore for 10 min. The animal was then placed in its home cage for 24 h. This retention interval was used to test long-term memory. In the second stage, one of the objects was replaced with a new one, and the mouse was again placed in the center of the arena for 5 min. Video recording of the mouse behavior was carried out using a recording device located above the analyzed object. The location of the mouse nose at a distance of no more than 2 cm from the object served as a criterion for the object exploration. To obtain the discrimination index, we calculated the ratio of the time spent on studying a new object to the total time of the objects’ exploration. The objects and the testing equipment were thoroughly cleaned with 70% ethanol after each animal.

### 2.8. Immunohistochemical Studies

On the seventh day of DS treatment, the animals were deeply anesthetized with isoflurane (Laboratories Kari-zoo, S.A., Barcelona, Spain) for brain extraction and immunohistochemistry using a rodent anesthesia vaporizer (VetFlo™, Kent Scientific Corporation, Torrington, CT, USA) equipped with a mouse mask. Anesthetized animals were transcardially perfused with 5 mL of PBS (~4 °C), pH 7.2. Both hemispheres were used for the study. After fixation, the brain was washed with PBS (pH 7.2) and embedded in paraffin blocks. Using a Leica RM 2245 microtome (Leica, Wetzlar, Germany), 10 µm thick sagittal sections were made. To block endogenous peroxidase activity, the slides were incubated in 0.3% hydrogen peroxide solution for 5 min. Blocking of non-specific binding of antibodies was performed by incubation with 5% BSA in PBS for 1 h. Further incubation was carried out with a solution of primary antibodies and secondary antibodies conjugated with horseradish peroxidase: anti-rabbit, 1:200, PI-1000-1; anti-mouse 1:200, PI-2000-1 (both from Vector Laboratories, San Francisco, CA, USA). Staining was performed using ImmPACT™ DAB peroxidase chromogen substrate (SK-4105, Vector Laboratories, San Francisco, CA, USA). The slides were then washed with 0.1 M PBS (pH 7.2), dehydrated, and mounted in VectaMount Permanent Mounting Medium (H-5000, Vector Laboratories). The following primary polyclonal rabbit antibodies were used: rabbit polyclonal antibodies to Iba-1 (1:500, ab108539, Abcam), rabbit monoclonal antibodies to S100β (1:1000, ab52642; Abcam), mouse monoclonal antibodies to PCNA (1:1000, ab29; Abcam), rabbit polyclonal antibodies to doublecortin (1:1000, ab18723, Abcam).

A Zeiss Axio Imager microscope equipped with an AxioCam 503 color camera and Axio-Vision software (Carl Zeiss, Oberkochen, Germany) was used to visualize slides. The image processing and analysis were performed using ImageJ software (NIH, Bethesda, MD, USA). Each micrograph was converted to an 8-bit image and the background was subtracted (rolling ball radius = 50). Measurements of the immunopositive staining area and the immunopositive cells’ density were performed in the CA1, CA3, and DG regions of the hippocampus on every sixth slice. The researcher, who was not aware of the sections’ identity, carried out the measurements and evaluation.

### 2.9. Enzyme-Linked Immunosorbent Assay

To determine the concentration of cytokines in the mouse hippocampus, the enzyme-linked immunosorbent assay (ELISA) was used. The animals were anesthetized with isoflurane and the hippocampi were quickly removed, frozen in liquid nitrogen, and stored at −80 °C. Both the right and left hippocampus were used for analysis. The sample homogenization was performed using a homogenizing buffer consisting of 100 mM Tris, pH 7.4, 150 mM NaCl, 1 mM EGTA and 1 mM EDTA, 1% Triton X-100, 0.5% sodium deoxycholate with a cocktail of protease inhibitors (cOmplete™, Sigma-Aldrich). The obtained homogenates were incubated on ice for 15 min, centrifuged (16,000× *g*, 30 min, +4 °C), and supernatants were collected. For protein concentration analysis, the BCA kit (Pierce, Rockford, IL, USA) was used. ELISA kits were used for the detection of TNF-α (ab208348) and IL-6 (ab46100), all from Abcam. The absorbance at 450 nm was measured using an iMark plate spectrophotometer (Bio-Rad, Hercules, CA, USA).

### 2.10. Statistical Analysis

The data are presented as mean ± standard error of the mean. We tested all data for normal distribution with the Shapiro–Wilk test. Because the data were normally distributed, they were subjected to statistical analysis using two-way ANOVA followed by Tukey’s post hoc test of multiple comparisons. For in vitro studies and lipid analysis, one-way ANOVA with post hoc Tukey test was used. A *p*-value < 0.05 was considered to indicate a statistically significant difference. In in vitro studies, a plate well was used as an analysis unit. During the Western blot, the number of samples per group was 4; during the Griess test, the number of samples per group was 6. For in vivo studies, an individual animal was used as the unit of analysis. For lipid analysis, behavioral tests, and ELISA, the number of animals per group was 8, for immunohistochemical studies the number of animals per group was 10. For each marker, 5 sections from one animal were analyzed. Thus, the number of sections per group was 50. During the ELISA, each sample was repeated 3 times, then the obtained values were averaged. In the Y-maze analysis, the number of experimental sessions per group (16 per group) was used as the unit of analysis. Microsoft Excel software (Microsoft, Tulsa, OK, USA) and GraphPad Prism 4 (GraphPad Software, San Diego, CA, USA) were used for statistical processing.

## 3. Results

### 3.1. Fatty Acid-Derived NAE Supplementation Reduces Proinflammatory Cytokines Production in LPS-Activated Microglial Cells

**Figure 3:**
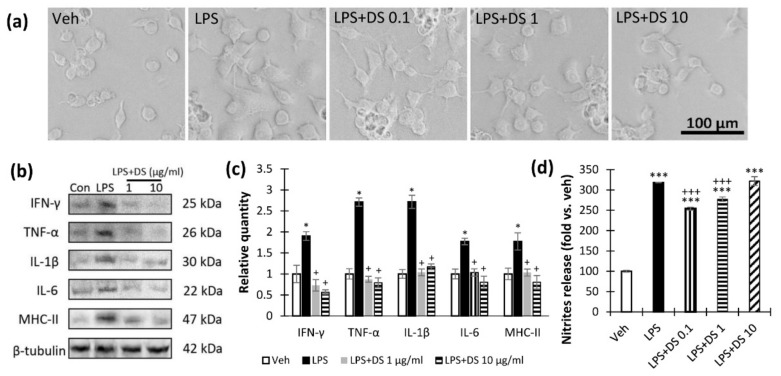
Effects of LPS and DS treatment on microglial morphology, proinflammatory cytokines, and NO production. (**a**) Representative images of SIM-A9 microglia cell culture after LPS (1 μg/mL) and DS (0.1, 1 and 10 μg/mL) treatment, scale bar—100 μm. (**b**) Western blot analysis of SIM-A9 microglia cell lysates (**a**) INFγ (25 kDa), TNFα (26 kDa), IL-1β (30 kDa), IL6 (22 kDa), and MHCII (47 kDa) were detected in LPS-activated SIM-A9 cell lysates with β-tubulin protein expression used as a loading control. Original uncropped images of blots are shown in Figure S1 in Supplementary Materials. (**c**) Relative quantity of INFγ, TNFα, IL-1β, IL6, and MHCII in SIM-A9 microglia cell lysates determined with Western blot, *n* = 4, * *p* < 0.001—compared to Veh, + *p* < 0.001—compared to LPS. (**d**) Nitrite production was detected with the Griess test, *n* = 6 per group. Data are presented as mean ± SEM, ^+++^ *p* < 0.001 — compared to LPS, ***p < 0.001 — compared to Veh.

At the first stage, we assessed the morphology of microglial cells after the lipopolysaccharides (LPS) and DS treatment. We observe LPS-mediated activation of microglial cells, expressed as an increase in their size and flattening. LPS treatment caused elongation and multiplication of the processes as well as gave the cells a fusiform shape. When the ethanolamine composition was added to the cells, a larger number of cells retained a rounded shape with a smaller number of processes (Figure 3a).

To reveal the anti-inflammatory activity of the study composition, we investigated the level of pro-inflammatory factors INFγ, TNFα, IL-1β, IL6, and MHCII after LPS (1 μg/mL) or DS (1 and 10 μg/mL) treatment (Figure 3b). As a result, we found a significant increase in the production of pro-inflammatory cytokines in the “LPS” group, while the addition of DS to the culture at concentrations of 1 and 10 μg/mL prevented these processes (*p* < 0.001). In addition, DS reduced IL6, MHCII, and TNFα below the “Veh” group (*p* < 0.001) (Figure 3c). The Griess test revealed an increase in LPS-induced nitrite production, while DS concentrations of 0.1 and 1 μg/mL prevented the increase in nitrite levels (Figure 3d).

### 3.2. Effect of DS Administration on Brain N-acylethanolamine Composition

**Figure 4:**
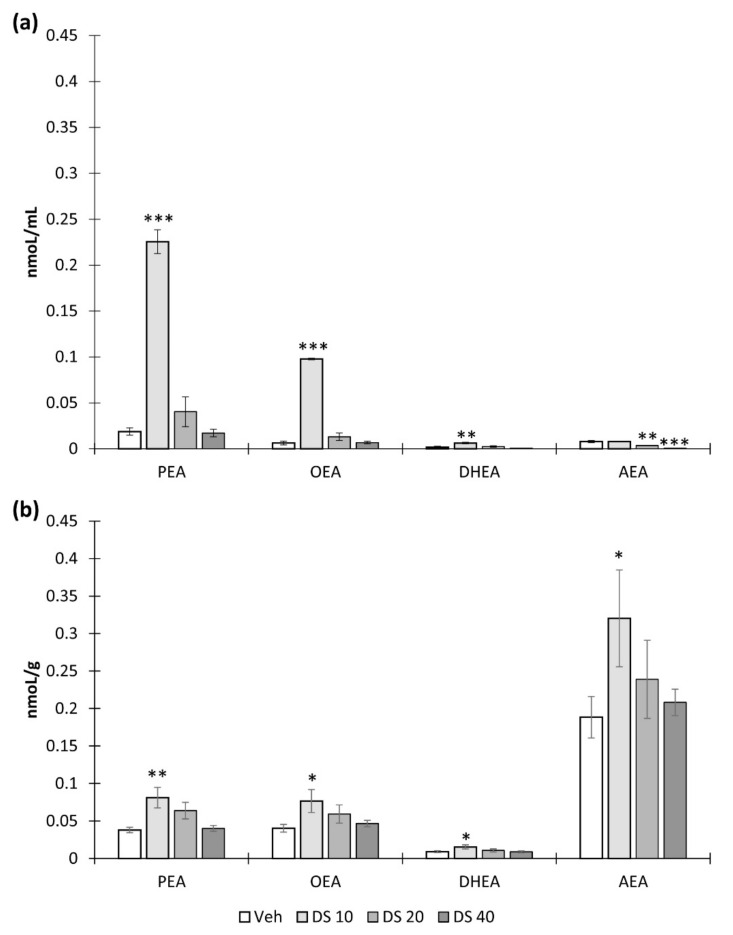
Effect of DS in doses 10, 20, and 40 mg/kg administered for 7 consecutive days on *N*-acylethanolamines concentration in plasm (**a**) and brain (**b**). Mean ± SEM, *n* = 8 (number of animals per group). One-way ANOVA with post hoc Tukey test, * *p* < 0.05, ** *p* < 0.01, *** *p* < 0.001, *—compared to Veh.

We started the in vivo block of DS biological activity studies with the quantification of brain *N*-acylethanolamines (NAE) composition after DS oral administration for 7 consecutive days. The results of this study allowed us to choose the optimal dose of DS for further experiments. Therefore, we found that the administration of DS at a dose of 10 mg/kg leads to a significant increase in the concentration of palmitoyl ethanolamide (PEA) (0.02 ± 0.004—“Veh” vs. 0.22 ± 0.012—“DS10”, *p* < 0.001), oleoyl ethanolamide (OEA) (0.006 ± 0.002—“Veh” vs. 0.09 ± 0.0007—“DS10”, *p* < 0.001), and docosahexaenoyl ethanolamide (DHEA) (0.002 ± 0.001—“Veh” vs. 0.006 ± 0.0008—“DS10”, *p* < 0.05). However, DS at a dose 20 mg/kg and 40 mg/kg significantly reduced plasma AEA concentrations (0.007 ± 0.001—“Veh” vs. 0.003 ± 0.0001—“DS20”, *p* < 0.01 and 0.0003 ± 0.0001—“DS40”, *p* < 0.001) (Figure 4a). We also obtained similar results by studying the concentration of NAE in the brain. Thus, only a concentration of 10 mg/kg significantly increased the content of PEA (0.03 ± 0.003—“Veh” vs. 0.08 ± 0.013—“DS10”, *p* < 0.01), OEA (0.04 ± 0.005—“Veh” vs. 0.07 ± 0.015—“DS10”, *p* < 0.05), DHEA (0.02 ± 0.0012—“Veh” vs. 0.002 ± 0.012—“DS10”, *p* < 0.05), and AEA (0.18 ± 0.02—Veh” vs. 0.32 ± 0.06—“DS10”, *p* < 0.05) within the brain (Figure 4b).

### 3.3. DS Administration Reverse LPS-Induced Hippocampal-Dependent Memory Impairment

At the next stage, we performed behavioral testing of animals treated with LPS or/and DS (10 mg/kg). LPS-induced neuroinflammation is a well-known and widely used model due to its availability and flexibility in the choice of dosing regimen to modulate the strength and duration of the pro-inflammatory response. Bacterial LPS are used to modulate both acute [39,40] and chronic neuroinflammation due to the presence of delayed effects of endotoxin administration [41,42,43]. A number of publications indicate that even a single administration of LPS to animals caused long-term cognitive consequences, as well as biochemical and morphological changes within the brain [42,43,44,45,46].

Since we chose the hippocampus as the area of interest, we had to investigate how hippocampus-dependent functions change during neuroinflammation and treatment. The most important of these functions are spatial working and long-term memory, which we studied using the Y-maze test and the “Novel object recognition” test.

**Figure 5:**
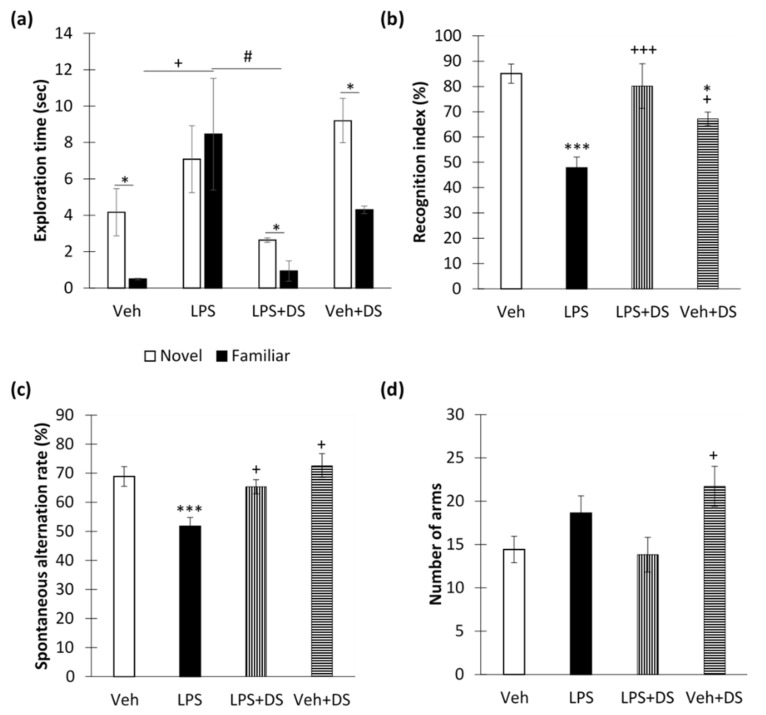
Behavioral effects of LPS and DS treatment. (**a**) Objects’ exploration time in novel objects recognition test, sec. (**b**) Recognition index in novel objects recognition test, %. Mean ± SEM, *n* = 8 (number of animals per group). (**c**) Spontaneous alternations rate in the Y-maze, %. (**d**) The number of arms passed through the Y-maze. Mean ± SEM, *n* = 16 (number of experimental sessions per group). Two-way ANOVA with post hoc Tukey test, ^#^ *p* < 0.05, * *p* < 0.05, *** *p* < 0.001; ^+^ *p* < 0.05, ^+++^ *p* < 0.001. *—compared to Veh, +—compared to LPS, # - compared to LPS + DS.

As expected, neuroinflammation led to impaired long-term memory, expressed as a decrease in the novel object recognition index (85.04 ± 3.78—“Veh” vs. 47.83 ± 4.22—“LPS”, *p* < 0.001) (Figure 5b) and an increase in the total exploration time of a familiar object (0.75 ± 0.07—“LPS” vs. 12.68 ± 2.76—“LPS + DS”, *p* = 0.025) (Figure 5a). However, DS administration prevented the LPS-mediated decrease in the recognition index (47.83 ± 4.22—“LPS” vs. 80.14 ± 8.82—“LPS + DS”, *p* < 0.001) (Figure 5a), and the time of familiar object examination decreased to the level of the “Veh” group (1.40 ± 0.83) (Figure 5b).

In addition, neuroinflammation led to a decrease in the spontaneous alternations rate in the Y-maze (*p* < 0.01). However, DS prevented the decrease in working memory score (51.72 ± 3.04—“LPS” vs. 65.31 ± 2.44—“LPS + DS”, *p* < 0.05). Two-way ANOVA showed a significant effect of LPS (F (3,64) = 14.70, *p* < 0.001) and DS (F (3,64) = 7.25, *p* = 0.009) (Figure 5c). It is surprising that locomotor activity, expressed as the number of arms passed through the Y-maze, did not change after LPS administration, but increased after DS administration to animals without neuroinflammation compared to the control (14.42 ± 1.54—“Veh” vs. 21.71 ± 2.31—“Veh + DS ”, *p* < 0.05) (Figure 5d).

### 3.4. PUFA Ethanolamides Composition Prevents Hippocampal Microglial Activation and Cytokine Production Increase

**Figure 6:**
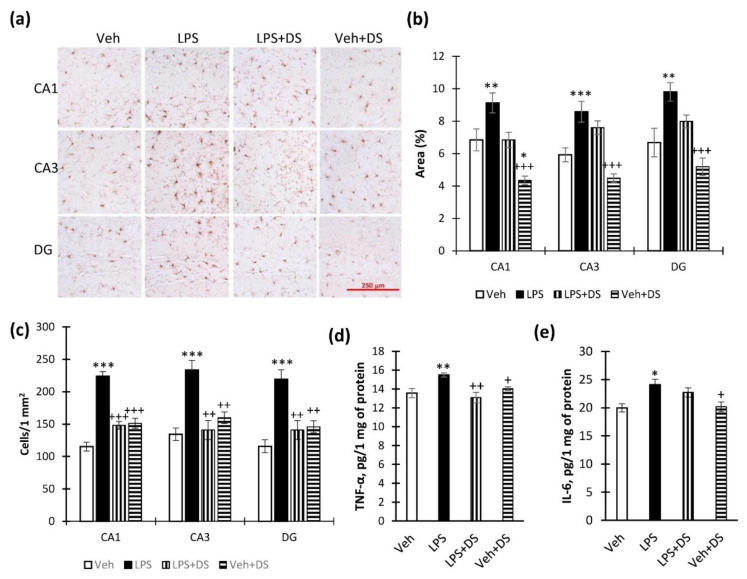
Effect of LPS and DS administration on hippocampal microglial activation and cytokine production. (**a**) Representative images of Iba-1+ microglial cells in CA1, CA3, and DG. (**b**) Histogram demonstrating the percentage of area covered by Iba-1+ staining in CA1, CA3, and DG regions. Mean ± SEM, *n* = 10 (number of animals per group). (**c**) Histogram demonstrating the number of Iba-1+ cells in CA1, CA3, and DG regions. Mean ± SEM, *n* = 10 (number of animals per group). (**d**) Production of TNF-α within the hippocampus after LPS and DS administration, determined by ELISA. (**e**) Production of IL-6 within the hippocampus after LPS and DS administration, determined by ELISA. Mean ± SEM, *n* = 8 (number of animals per group). Two-way ANOVA with post hoc Tukey test, * *p* < 0.05, ** *p* < 0.01, *** *p* < 0.001; ^+^ *p* < 0.05, ^++^ *p* < 0.01, ^+++^ *p* < 0.001. *—compared to Veh, +—compared to LPS.

At the next stage, the anti-inflammatory activity of DS was evaluated in vivo. Iba-1 is considered to be a classic marker for assessing microglial activity in neuroinflammation and is used in various pharmacological studies [47]. As expected, LPS administration to mice increased the Iba-1 immunopositive microglia (Figure 6a) percentage within the CA1 (6.84 ± 0.67—“Veh” vs. 9.12 ± 0.61—“LPS”), CA3 (5.92 ± 0.43“Veh” vs. 8.57 ± 0.64—“LPS”), and DG (6.68 ± 0.87—“Veh” vs. 9.79 ± 0.57—“LPS”) areas. However, DS reversed this effect in the CA1 region (9.12 ± 0.61—“LPS” vs. 6.83 ± 0.47—“LPS + DS”) and DG (9.79 ± 0.57—“LPS” vs. 7.98 ± 0.39—“LPS + DS”), while in the CA3 region, DS did not prevent a decrease in the Iba-1-positive microglia percentage (Figure 6b). Two-way ANOVA showed the effect of LPS (F (3,40) = 21.98, *p* < 0.001) and DS (F (3,40) = 22.07, *p* < 0.001) in CA1; LPS (F (3,40) = 41.71, *p* < 0.001) and DS (F (3,40) = 7.37, *p* = 0.01) in CA3; LPS (F (3,40) = 26.91, *p* < 0.001) and DS (F(3,40) = 8.43, *p* = 0.06) in DG. Similar results were obtained when counting Iba^+^-cells in the CA1, CA3, and DG regions. However, the number of immunopositive cells in the “LPS + DS” group of the studied regions was significantly lower than in the “LPS” group (*p* < 0.001) (Figure 6c). In the case of neuroinflammation, along with quantitative changes in the population of microgliocytes, qualitative changes also occur, which is expressed in morphology changes, namely, the retraction processes, an increase in the cell bodies’ area, and the gradual acquisition of an amoeboid shape. The discrepancies in the results when using different approaches to counting are associated with these features of microgliocytes. The DS treatment prevents an increase in the number of microgliocytes to a greater extent, and, to a lesser extent, morphological changes. Since activated microglia produce pro-inflammatory cytokines, we examined the hippocampal production of TNF-α and IL-6 within the hippocampus. As a result, we found that LPS increased TNF-α production, while DS inhibited this effect (15.49 ± 0.22—“LPS” vs. 13.09 ± 0.55—“LPS + DS”, *p* < 0.01). Two-way ANOVA showed the effect of LPS (F (3,32) = 6.56; *p* = 0.015), DS (F (3,32) = 4.61; *p* = 0.053), and LPS/DS interaction (F (3,40) = 31.63; *p* < 0.001) (Figure 6d). At the same time, DS did not interfere with the LPS-mediated increase in IL-6 production (24.09 ± 0.95—“Veh” vs. 22.71 ± 0.79—“LPS”, *p* > 0.05). Two-way ANOVA showed the effect of LPS (F (3,32) = 17.65, *p* < 0.001), but not DS (Figure 6e).

### 3.5. DS Administration Prevents Hippocampal S100β Production

Astrocyte activation occurs as a response to pathological conditions during neuroinflammation and, as a rule, takes place followed by microglia activation, although it can also develop through independent mechanisms [48]. As a marker for astroglial activity assessment, we chose the cytosolic protein S100β. This protein plays an important role in mediating innate and acquired immune responses that contribute to chronic inflammatory disease development [49].

**Figure 7:**
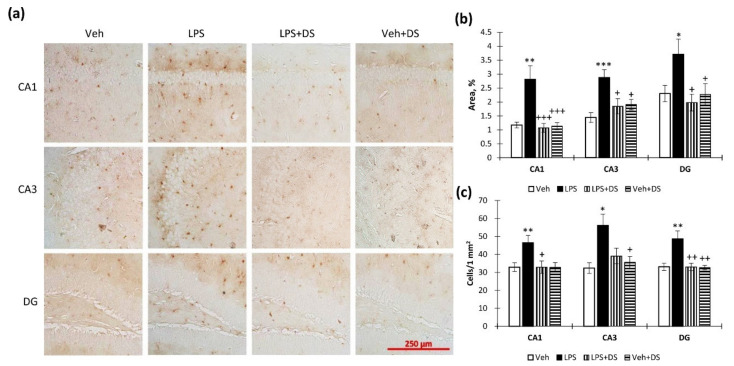
Effect of LPS and DS administration on hippocampal S100β immunoreactivity. (**a**) Representative images of S100β^+^ microglial cells in CA1, CA3, and DG. (**b**) Histogram demonstrating the percentage of area covered by S100β^+^ staining in CA1, CA3, and DG regions. Mean ± SEM, *n* = 10 (number of animals per group). (**c**) Histogram demonstrating the number of S100β^+^-cells in CA1, CA3, and DG regions. Mean ± SEM, *n* = 10 (number of animals per group). Two-way ANOVA with post hoc Tukey test, * *p* < 0.05, ** *p* < 0.01, *** *p* < 0.001; ^+^ *p* < 0.05, ^++^ *p* < 0.01, ^+++^ *p* < 0.001. *—compared to Veh, +—compared to LPS.

We found an increase in hippocampal S100β immunoreactivity after LPS administration. DS administration caused the prevention of LPS-induced S100β upregulation (Figure 7a) in CA1 (2.81 ± 0.48—“LPS” vs. 1.06 ± 0.16—“LPS + DS”, *p* < 0.001), CA3 (2.87 ± 0.27—“LPS” vs. 1.84 ± 0.27—“LPS + DS”, *p* < 0.05), and DG (3.70 ± 0.54—“LPS” vs. 1.97 ± 0.30—“LPS + DS”, *p* < 0.05) (Figure 7b). Two-way analysis of variance showed the effect of LPS (F(3,40) = 12.63, *p* < 0.001), DS (F(3,40) = 25.80, *p* < 0.001)—CA1; (F(3,40) = 12.24, *p* < 0.001), DS (F(3,40) = 10.13, *p* = 0.01)—CA3; LPS (F(3,40) = 2.89, *p* = 0.028), DS (F(3,40) = 10.91, *p* = 0.002)—DG. Similar results were obtained when counting S100β^+^-cells in the CA1, CA3, and DG regions (Figure 7c).

### 3.6. Fatty Acids Ethanolamides Composition Reverse Hippocampal Neurogenesis Deterioration

**Figure 8:**
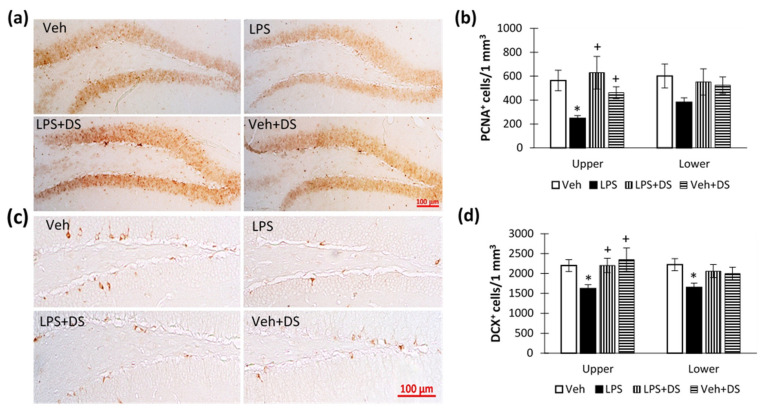
Number of PCNA- and doublecortin-positive neurons in the subgranular layer of the hippocampal DG SGZ. (**a**) Representative images of PCNA-positive neurons in the DG SGZ. (**b**) Plot showing the number of PCNA-positive neurons in DG SGZ, *n* = 10 (number of slices per group), * *p* < 0.05, ^+^ *p* < 0.05. (**c**) Representative images of doublecortin-positive neurons in the DG SGZ. (**d**) Plot showing the number of doublecortin-positive neurons in DG SGZ, *n* = 10 (number of slices per group), * *p* < 0.05, ^+^ *p* < 0.05.

Since adult hippocampal neurogenesis is the basis for the hippocampus-dependent functions and is impaired during neuroinflammation, we studied the effects of inflammation and treatment on the number of PCNA- and doublecortin-positive neurons in the hippocampal dentate gyrus subgranular zone (DG SGZ) (Figure 8a,c).

As expected, systemic LPS administration contributed to a decrease in the number of PCNA- and doublecortin-positive cells in the DG SGZ. Significant changes in the number of PCNA-positive cells were observed only in the SGZ of the DG upper blade. There is a well-defined difference in the number of PCNA-positive cells in LPS-treated groups with and without treatment (246.90 ± 23.73—“LPS” vs. 628.66 ± 136.08—“LPS + DS”, *p* < 0.05) (Figure 8b). In addition, LPS treatment contributed to a decrease in the number of doublecortin-positive neurons in the SGZ of the hippocampal upper blade. However, the composition of fatty acids ethanolamides prevented this effect (1619.7 ± 100.80—“LPS” vs. 2201.35 ± 181.14—“LPS + DS”, *p* < 0.05) (Figure 8d). Two-way analysis of variance showed the effect of LPS (F (3,40) = 8.16; *p* < 0.005), DS (F (3,40) = 4.61; *p* < 0.033), and LPS/DS interaction (F (3152) = 4.42; *p* < 0.037).

## 4. Discussion

This work is focused on the study of the biological effects of the lipid composition containing fatty acid-derived *N*-acylethanolamines obtained from squid *Berryteuthis magister* processing by-products. Our work is based on the study of the neurotropic pharmacological activity of the tested lipid composition with a focus on the hippocampus. The hippocampus is a part of a limbic system responsible for the formation of spatial memory necessary for navigation, episodic memory, as well as memory consolidation, and emotion formation [50]. As a model for studying the effect of DS on the hippocampus, we chose the model of LPS-induced neuroinflammation because hippocampus-dependent functions are extremely sensitive to the influence of pro-inflammatory agents [51]. In addition, based on more recent reports describing the anti-inflammatory activity of fatty acids-derived NAEs, we suggested the anti-inflammatory activity of the studied dietary supplement [26,52,53,54]. N fatty acids-derived NAEs, e.g., *N*-docosahexaenoyl ethanolamine (DHEA), *N*-eicosapentaenoyl ethanolamine (EPEA), *N*-stearoyl ethanolamine (SEA), *N*-palmitoyl ethanolamine (PEA), and *N*-oleoyl ethanolamine (OEA), are natural lipid signaling molecules. The natural sources of these compounds are very diverse and can include both plant and animal organisms. Oral DS increased the content of DHEA both in plasma and in the brain, but, unfortunately, we were not technically able to quantify SEA and EPEA. Even though the three main components of our dietary supplement are SEA, DHEA, and EPEA, oral administration of this mixture led to a significant increase in the concentration of other NAEs both in blood plasma and in the brain. This may indicate that exogenous NAEs serve as precursors for the synthesis of metabolites required by the body. The main enzyme involved in NAE hydrolysis is fatty acid amide hydrolase (FAAH) [22], which cleaves NAE into fatty acids and ethanolamine [54]. Hydrolysis products of NAE are involved in the biosynthesis of phosphatidylethanolamine and *N*-acylphosphatidylethanolamine, which are precursors of *N*-acylethanolamines. However, not all NAEs have the same affinity for FAAH. An increase in DHEA concentration can inhibit FAAH activity against other NAEs due to competitive exclusion from substrate binding [25]. For example, Bisogno et al., 1999 [55] showed that synaptamide inhibits AEA hydrolysis, although not as strongly as AEA itself.

Thus, the observed anti-inflammatory effect of DS can be based on the increase in total brain NAE content. The biological effects of *N*-acylethanolamines are diverse and, in addition to anti-inflammatory activity, include other properties. For example, the anti-inflammatory, analgesic, and neuroprotective effects of PEA are well documented [56], as well as the anti-inflammatory and metabotropic effects of OEA [14,57]. DHEA occupies a special position among these metabolites due to its neurotropic activity. Even the term “synaptamide” reflects the synaptogenic activity of this compound [53,58]. Paradoxically, only the lowest concentration of DS (10 mg/kg) resulted in a significant NAE accumulation in both plasma and brain. It is also interesting that high doses of DS (20 and 40 mg/kg) resulted in a decrease in AEA plasma levels compared to Veh-treated animals. These data are consistent with previous findings where a DHA-enriched diet resulted in a decrease in plasma and tissue arachidonic acid (AA) derivatives [59]. The decrease in the n-6/n-3 ratio can be explained by enzymatic competition in the biosynthesis of different NAE species. It is expected that high concentrations of n-3 derivatives may reduce the content of AA metabolites [60].

Numerous studies show that even a single administration of LPS endotoxin to experimental animals leads to long-term morphological and behavioral consequences [61,62], which probably also occur due to severe infectious diseases in humans. Thus, accelerating the resolution process in chronic neuroinflammation may be an effective strategy to deal with the cognitive consequences of severe generalized inflammatory processes. A therapy aimed at stimulating the endogenous production or exogenous administration of pro-resolving lipid mediators appears to be very promising. In our study, the pro-inflammatory agent LPS led to a disruption in hippocampal functioning, which was expressed in the deterioration of hippocampal-dependent working spatial and long-term memory. Similar results were obtained earlier, and they are covered in the literature [63,64]. Our experimental data confirmed our hypothesis that the studied DS reverses LPS-induced memory impairment. Behavioral findings served as indirect evidence of the DS anti-neuroinflammatory activity. Moreover, these data were confirmed by immunohistochemical and enzyme immunoassay studies of the glial markers and pro-inflammatory cytokines production within the hippocampus. The DS treatment prevented an increase in Iba-1^+^ microglia and S100β^+^ astroglia hippocampal activity, and also reduced the production of TNF-α. However, the drug did not reduce the LPS-induced increase in IL-6 production. This difference in effects is difficult to explain, but may be because inflammatory cytokines are upregulated in LPS-stimulated microglial cells through different signaling cascades [65]. It is worth noting that in this study, only a moderate increase in the production of pro-inflammatory cytokines was observed, which is presumably related to the time course of the neuroinflammatory process. Our preliminary experiments show that the most powerful release of pro-inflammatory cytokines is observed in the first hours after endotoxin administration, which is also confirmed in other studies [44,45,46]. Taken together, these studies indicate that cytokine production is highly variable with LPS dosing regimen, the strain of mice, and other conditions. Even though there is a gradual decrease in the pro-inflammatory factors’ production after the peak of their release, this response is sufficient to mediate changes in neuronal plasticity and impair memory. The results of in vivo experiments are also confirmed by our data obtained in in vitro tests. We observed a marked decrease in the LPS-induced production of pro-inflammatory factors INFγ, TNFα, IL-1β, IL6, and MHCII in SIM-A9 microglia cell and nitrites, which are actively produced during neuroinflammation by activated microglia [66].

Given the dual role of the neuroinflammatory response, an important question is whether the suppression of neuroinflammation will cause negative consequences. Researchers agree that with the timely resolution of the neuroinflammatory process, the negative consequences are gradually leveled [67]. The dual role of neuroinflammation is that it simultaneously activates protective processes in the brain, and a destructive effect is observed, especially with a long course [68]. For example, the pro-inflammatory cytokine IL-1β can have both a neurotoxic effect, stimulating the entry of calcium into neurons, and promote the synthesis of survival-promoting factors by astrocytes [69,70,71]. The pro-inflammatory cytokine IL-6 is essential for normal brain development, but it is also involved in the pathogenesis of neuroinflammation [72]. Additionally, TNF-α can increase neurotoxicity by inhibiting glutamate uptake [73] and stimulating oxidative stress [74], but at the same time, activates the neurotrophic factors’ production [75]. One of the negative consequences of the neuroinflammatory response and the massive release of pro-inflammatory cytokines is the impairment of hippocampal neurogenesis [52], which is known to be the fundamental basis for hippocampal functioning throughout life [76]. In vivo experiments have shown the involvement of IL-1β/NF-κB signaling in the reduction of adult hippocampal neurogenesis [77,78,79], while IL-1 and TNF signaling blocking positively regulates neural progenitor proliferation [80,81]. Regulation of glial activity and release of cytokines probably underlies the DS pro-neurogenic effect and is realized through several signaling pathways. An increase in the brain endocannabinoid AEA concentration after DS administration can contribute to the activation of CB receptors and, as a result, the inflammatory response suppression. Unlike AEA, NAE derivatives of other fatty acids, including OEA, PEA, and DHEA, act predominantly through CB-independent mechanisms, while being powerful inflammation suppressors. For example, in a comparative study by Meijerink et al., (2011) [25], DHEA and EPEA showed to be the most potent inhibitors of NO release in a dose-dependent manner. The anti-inflammatory mechanisms of n-3 derived NAEs are mediated predominantly through nuclear peroxisome proliferator-activated receptor-α (PPAR-α), which mediates many anti-inflammatory effects, as well as through a decrease in the production of pro-inflammatory factors by suppressing NF-kB [12] and interfering with the ERK1/2-dependent signaling pathway [14]. There are specific receptors for NAE through which the inflammatory response is suppressed. For example, the GPR110 and GPR55 receptors enhance cAMP-dependent signaling in microglia and innate peripheral immune cells, followed by phosphorylation of protein kinase A (PKA) and the cAMP response element binding protein (CREB) [59]. Oxidized metabolites of DHEA can activate CB2 receptors, providing anti-inflammatory and tissue-protective effects [82]. However, it remains to be seen whether the effect of the DS is predominantly localized in the brain or whether the observed effects are due to the suppression of peripheral inflammation. Cytokines released in LPS treatment are formed in the periphery and migrate to the brain, bypassing the blood–brain barrier (BBB). LPS can increase BBB permeability, which in turn leads to the release of cytokines by brain endothelial cells [83]. Taking into account previous works investigating the anti-inflammatory activity of NAE in peripheral tissues [57,84], we can assume that the neuroinflammation suppression is partially mediated by peripheral effects. In addition to anti-inflammatory activity, individual NAEs also have specific activities, for example, DHEA promotes the development of hippocampal neurons [22]. PEA, in addition to being anti-inflammatory, has analgesic activity [85], and OEA promotes weight loss [20,21]. In general, the specific action of the individual components of the NAE complex provides a wide therapeutic application of the studied dietary supplement.

## 5. Conclusions

In this study, we have shown that a dietary supplement consisting of a fatty acid-derived NAEs mixture suppresses the neuroinflammatory response by reducing glial activation and pro-inflammatory cytokine release positively regulates neural progenitor proliferation and attenuates hippocampal-dependent memory impairment.

## Data Availability

The datasets used and analyzed during the current study are available from the corresponding author on reasonable request.

## Supplementary Materials

The following supporting information can be downloaded at: https://www.mdpi.com/article/10.3390/nu14183879/s1, Figure S1: Original uncropped images of blots.
