# Supplementary figures and images for: Fatty Acid-Derived N-acylethanolamines Dietary Supplementation Attenuates Neuroinflammation and Cognitive Impairment in LPS Murine Model
Source: Nutrients. 2022 Sep 19;14(18):3879. doi: 10.3390/nu14183879 (PMC9504857; doi:10.3390/nu14183879)

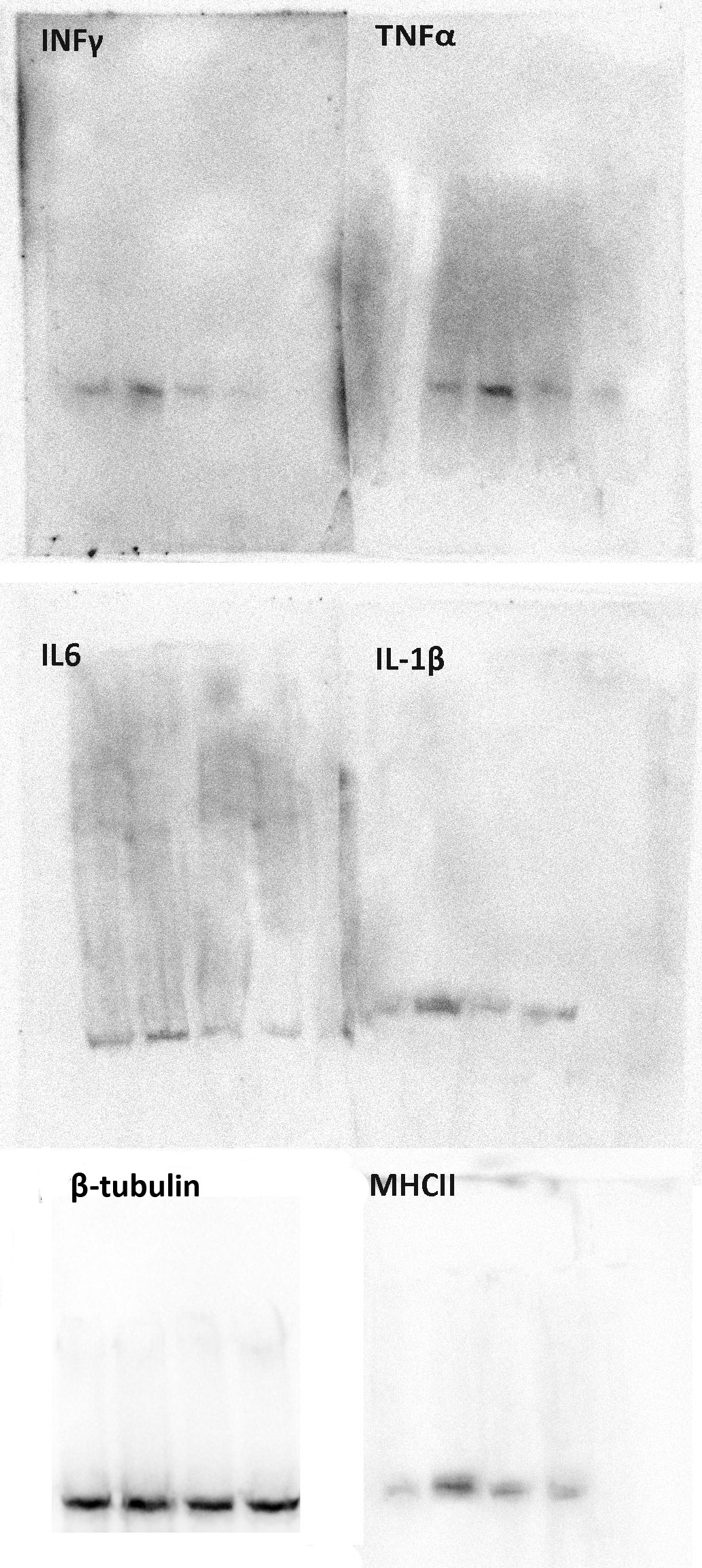

Supplement: Supplementary file 1 [file nutrients-14-03879-s001.zip › nutrients-1865714-Figure S1.jpg]
